# Effect of PKC inhibitor on experimental autoimmune myocarditis in Lewis rats

**DOI:** 10.18632/oncotarget.17018

**Published:** 2017-04-10

**Authors:** Chunlian Zhong, Yang Wu, He Chang, Chunxiao Liu, Li Zhou, Jun Zou, Zhi Qi

**Affiliations:** ^1^ Department of Basic Medical Sciences, Medical College of Xiamen University, Xiang’an Nan Lu, Xiamen, China; ^2^ Xiamen Cardiovascular Hospital, Medical College of Xiamen University, Xiamen, China

**Keywords:** myocarditis, PKC signaling, PKC inhibitor, apoptosis, inflammation

## Abstract

Myocarditis is a major cause of sudden, unexpected death in young people. However, it is still one of the most challenging diseases to treat in cardiology. In the present study, we showed that both expression level and activity of PKC-α were up-regulated in the rat heart of experimental autoimmune myocarditis (EAM). Intraperitoneal administration of PKC inhibitor (Ro-32-0432) at the end of the most severe inflammation period of EAM still significantly reduced the EAM induced expression of failure biomarkers. Furthermore, Ro-32-0432 reduced the ratio of Bax/Bcl-2 and suppressed the expression of cleaved caspase-3, both of which were increased in the heart of the EAM rats, suggesting an anti-apoptotic role of Ro-32-0432. Besides, Ro-32-0432 suppressed EAM-induced cardiac fibrosis and release of pro-inflammatory cytokines IL-1β and IL-17. These results suggest that inhibition of PKC may serve as a potential therapeutic strategy for the treatment of myocarditis.

## INTRODUCTION

Acute myocarditis is a multifaceted disorder characterized by an unpredictable clinical course which ranges from asymptomatic, incidentally discovered forms, to cases with fulminant course and adverse outcome [1]. It is a major cause of sudden, unexpected death in young people [2, 3]. It has been recognized that viruses, bacteria, protozoa, systemic diseases, autoimmune diseases, drugs and toxins are associated with the development of this disease. However, as a non-familial form of heart muscle disease [4], the etiology of myocarditis in any given patient often remains unknown [5, 6]. Even though it has been reported that patients with giant cell myocarditis can be successfully immunosuppressed [7], immunotherapy and specific antiviral treatment have yet to demonstrate definitive clinical efficacy for other types of myocarditis [8]. Thus, myocarditis is still one of the most challenging diseases to diagnose and treat in cardiology [8].

Protein kinase C (PKC), a family of serine-threonine protein kinase enzymes, regulates a number of cardiac responses [9]. Overexpression of PKC-α decreases contractility of the heart, while its knockout enhances contractility [10]. Overexpression of PKC-β causes hypertrophy and cardiac dysfunction [11]. PKC family members have also been demonstrated to play important roles in pathogenesis of many heart diseases, including heart failure [9, 12], myocardial infarction [13, 14], ischemia-reperfusion injury [15], myocardial hypertrophy [16], dilated cardiomyopathy [17] and diabetic cardiomyopathy [18]. Increased expression and activation of select PKC isoforms have been observed in a number of cardiac diseases, such as heart failure [9]. Thus, inhibition of PKC-α has been shown to be cardioprotective [12] and to improve myocardial contractility [10]. PKC-β inhibitor improves cardiac function in a porcine heart failure model [14]. Inhibition of PKC-ε suppresses chronic inflammation in murine cardiac transplantation model [19] and attenuates hypertension-induced heart failure [20]. Inhibition of protein kinase C α/β enhances cardiac contractility and attenuates heart failure [21]. PKC-δ activation mediates much of the acute injury induced after transient myocardial ischemia [22]; its inhibition reduces myocardial infarction induced microvascular dysfunction [23] and protects the heart from ischemia-reperfusion injury [15]. Therefore, PKC family members have been a focus of drug discovery and a therapeutic target for many kinds of diseases [24].

Based on these findings, we hypothesized that inhibition of PKC signaling may affect myocarditis as well. Experimental autoimmune myocarditis (EAM) is a well-known myocarditis model in rats, which mimics human myocarditis in the acute and chronic phases [25, 26]. It has been reported that the pathogenesis of both human giant-cell myocarditis and viral myocarditis resembles that of EAM [27]. Therefore, we investigated the effect of a PKC blocker on the EAM rats and the underlying cellular mechanism.

## RESULTS

### Expression and activity of PKC isoforms in the heart of EAM rats

**Figure 1:**
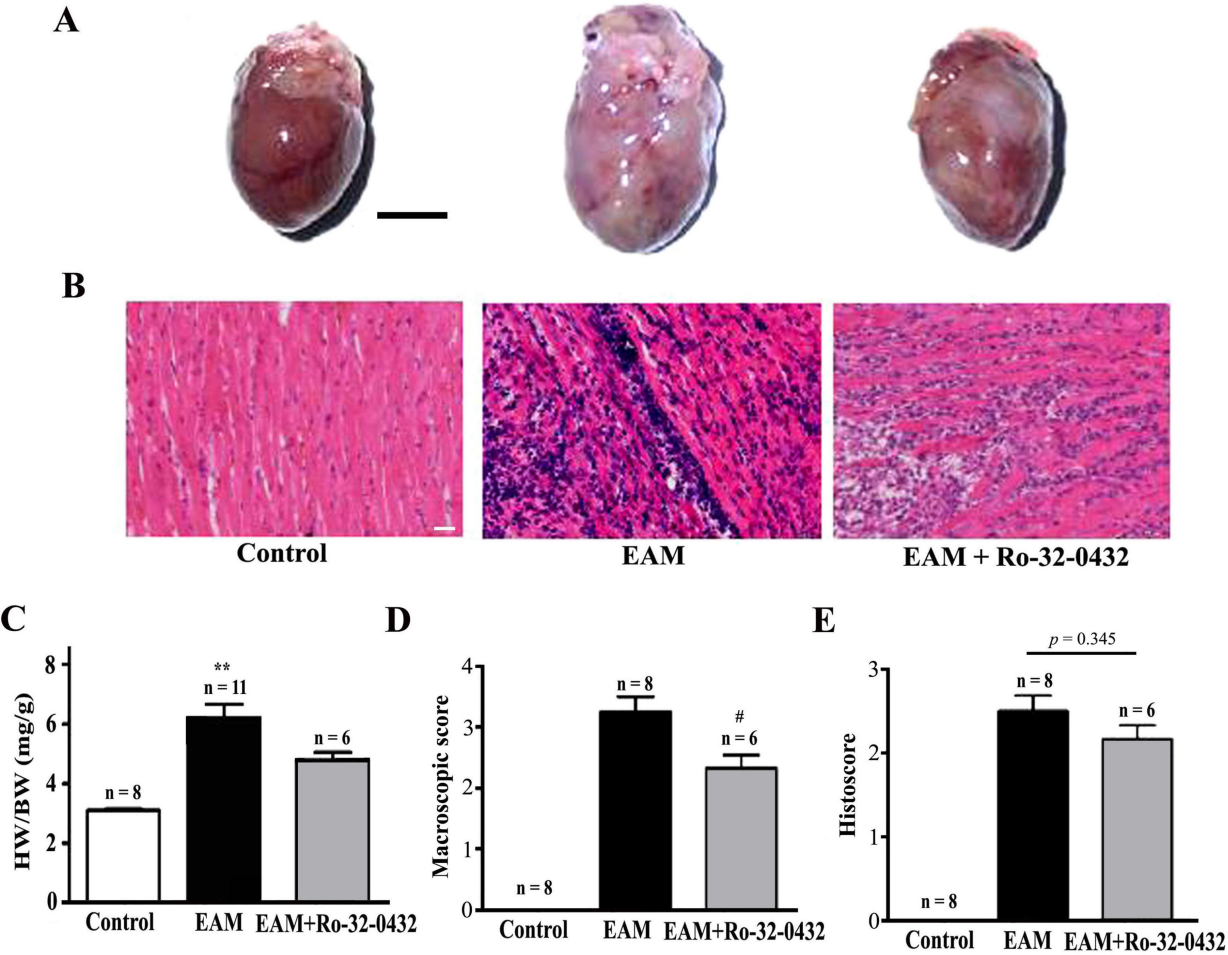
Histopathological analysis on the heart of EAM and Ro-32-0432 treated EAM rats (**A** and **B**) Representative HE staining of the whole heart (A, Bar = 5 mm) and heart section (B, Bar = 20 μm) of the control, EAM and Ro-32-0432 treated EAM groups. (**C**) The ratio of heart weight to body weight (HW/BW). (**D** and **E**) Histopathological examination of the hearts in different groups were shown by macroscopic score (D) and histoscore (E). **p < 0.01 vs. control; ^#^*p* < 0.05 vs. EAM.

**Figure 2:**
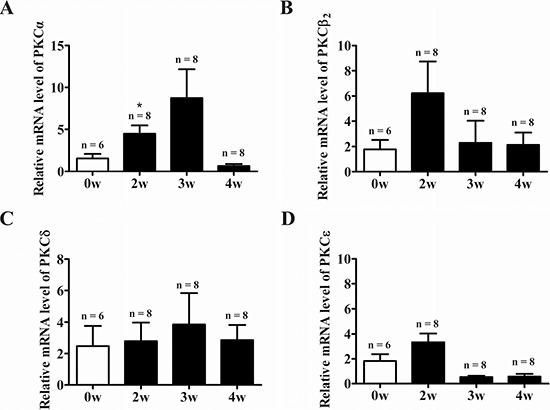
PKC family gene expression in the heart of EAM rats (**A**–**D**) Time course of relative mRNA expression level of the PKC isoforms in the heart normalized against GAPDH during progression of EAM: PKC-α (A), PKC-β2 (B), PKC-δ (C), and (D) PKC-ε. *p < 0.05 vs. Control.

**Figure 3:**
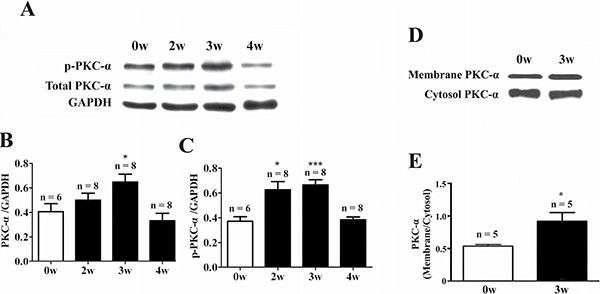
Up-regulation of PKC-α protein expression and activity in the heart of EAM rats (**A**–**C**) Western blot analysis of total PKC-α and phosphorylated PKC-α (p-PKC-α) in the heart of control and EAM rats. (**D** and **E**) Western blot analysis of membrane and cytosolic fractions of PKC-α in the heart of control and EAM rats. Level of p-PKC-α and total PKC-α were normalized to GAPDH. *p < 0.05 vs. control; **p < 0.01 vs. control; ***p < 0.001 vs. control.

At week 3 of EAM, the heart was markedly enlarged accompanied by the appearance of discolored surface (Figure 1A, middle panel). Compared to the control (Figure 1A, left panel), prominent infiltration of inflammatory cells into the myocardium could be observed in the transverse sections of cardiac ventricles (Figure 1B, middle panel). Meanwhile, the HW/BW (Figure 1C), macroscopic score (Figure 1D) and histoscore (Figure 1E) of EAM rats were significantly increased. Interestingly, among the PKC isoforms tested (PKC-α, PKC-β2, PKC-δ and PKC-ε), only the mRNA expression level of the PKC-α was significantly increased in the heart of EAM rats at week 2 (Figure 2A–2D). Furthermore, protein expression level of PKC-α was significantly elevated at week 3 (Figure 3A and 3B). To investigate whether activity of PKC was increased in the EAM rats, we assessed the phosphorylation of PKC-α. We found that phosphorylation of PKC-α at Thr 497, which reflects the activation of PKC-α [28, 29], was increased in EAM rats (Figure 3A and 3C) from week 2 to week 3. To further confirm the increase in PKC-α activity in EAM, we measured the translocation of PKC from the cytoplasm to the membrane, which has been used to indicate PKC activation [30, 31]. Western blotting analysis revealed that the membrane/cytosol ratio was significantly increased in EAM group at week 3 (Figure 3D and 3E). These results indicated that the activity of PKC-α was upregulated in the EAM.

### PKC inhibitor down-regulates biomarkers of heart failure in EAM rats

**Figure 4:**
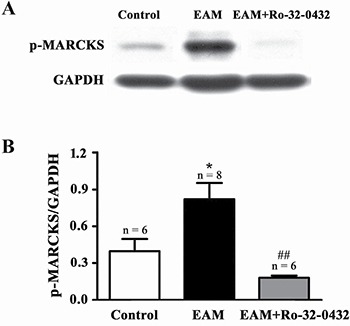
Ro-32-0432 suppresses EAM-induced activation of PKC in the heart (**A** and **B**) Representative Western blot (A) and statistical summary (B) on the phosphorylation of MARCKS (p-MARCKS) in control, EAM and Ro-32-0432 treated EAM groups. Level of p-MARCKS was normalized to GAPDH. *p < 0.05 vs control; ^##^*p* < 0.01 vs. EAM.

Next, we explored the possibility that blockade of PKC signaling by Ro-32-0432, an inhibitor of the classic PKC isoforms (especially PKC-α) [21], from week 2 of EAM may have a therapeutic potential against myocarditis. Previously, various dosages of Ro-32-0432, ranging from 1–200 mg/kg [32–34], have been used in *vivo* to effectively inhibit PKC. We intraperitoneally administrated Ro-32-0432 at a dosage of 1 mg/kg to the rats every two days from day 14 to day 18 of EAM, since our preliminary experiment suggested that this dosage was effective to reduce EAM-induced increase in the ratio of HW/BW. To test whether Ro-32-0432 could inhibit PKC activity in EAM, we investigated the effect of Ro-32-0432 on phosphorylation of myristoylated alanine-rich C kinase substrate (MARCKS) protein, a major PKC target protein [35, 36]. We found that Ro-32-0432 significantly inhibited the EAM-induced increase in the phosphorylation of the MARCKS protein at Ser158, which has been used as a marker for PKC activation [35] (Figure 4A and 4B). Thus, Ro-32-0432 could inhibit the EAM-induced upregulation of the PKC activity.

**Figure 5:**
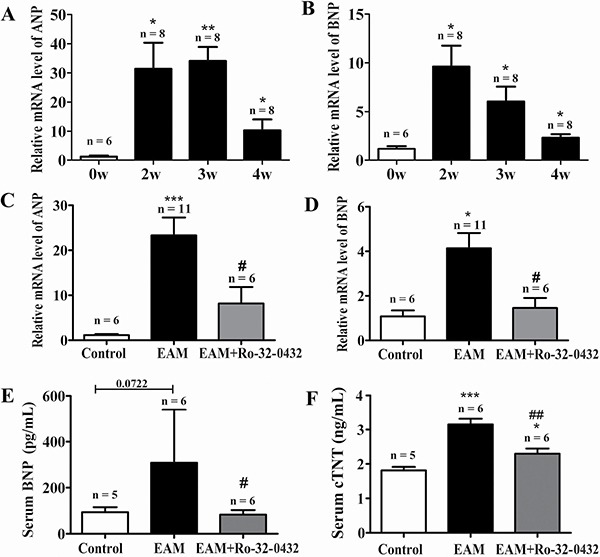
Ro-32-0432 suppresses heart failure biomarkers in the heart of EAM rats (**A** and **B**) Time course of relative mRNA levels of both ANP (A) and BNP (B) during progression of EAM. (**C** and **D**) Relative mRNA levels of both ANP (C) and BNP (D) measured at week 3 of EAM for control, EAM and Ro-32-0432 treated EAM groups. Relative mRNA levels were normalized against that of GAPDH. (E and F) Protein concentration of BNP (**E**) and cTnT (**F**) in the serum measured at week 3 of EAM for control, EAM and Ro-32-0432 treated EAM groups. *p < 0.05 vs. control; **p < 0.01 vs. control; ***p < 0.001 vs. control; ^#^*p* < 0.05 vs. EAM; ^##^*p* < 0.01 vs. EAM.

From week 2 of EAM, the mRNA level of both ANP and BNP, two of the diagnostic and predictive biomarkers of heart failure [37], were significantly increased compared with the control group (Figure 5A and 5B). Ro-32-0432 reduced the relative mRNA level of ANP from 23.3 ± 3.9 to 8.2 ± 3.7 (Figure 5C) and that of BNP from 4.1 ± 0.7 to 1.5 ± 0.4 (Figure 5D) at week 3. Furthermore, protein level of BNP (Figure 5E) and cardiac troponin T (cTnT, Figure 5F) in the serum, which were increased in the EAM rats, were reduced by Ro-32-0432. In good agreement with this result, HW/BW, which was 3.1 ± 0.4 for control group, was significantly reduced from 6.2 ± 0.5 of the EAM group to 4.8 ± 0.3 for that of the Ro-32-0432 treated group (Figure 1C) and the area of the discolored surface judged by the macroscopic score was significantly reduced as well (Figure 1D). These results implied that inhibition of PKC signaling could alleviate the myocarditis induced heart damage.

### PKC inhibitor suppresses apoptosis of myocardial cells

**Figure 6:**
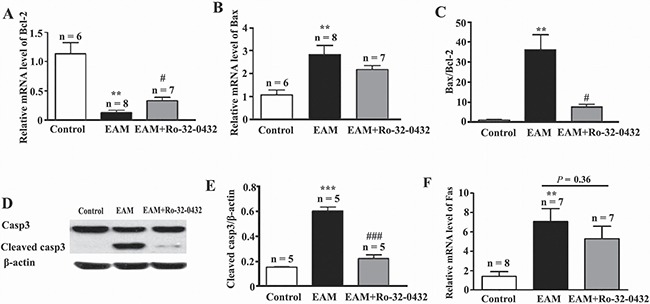
Ro-32-0432 suppresses apoptosis of myocardial cells in the heart of EAM rats (**A**–**C**) Relative mRNA expression of myocardial Bcl-2 (A), Bax (B) and the ratio of Bax to Bcl-2 (C) in the control, EAM and Ro-32-0432 treated EAM rats. (**D**) Representative western blot image of protein expression levels of caspase-3 (Casp3), cleaved caspase-3 (Cleaved casp3) and β-actin in the control, EAM and Ro-32-0432 treated EAM rats. (**E**) Relative protein expression level of cleaved caspase 3 in the heart of control, EAM and Ro-32-0432 treated EAM rats. Level of cleaved caspase 3 was normalized to β-actin. (**F**) Relative mRNA expression of myocardial Fas in the control, EAM and Ro-32-0432 treated EAM rats. **p < 0.01 vs control; ***p < 0.001 vs. control; ^#^*p* < 0.05 vs. EAM; ^###^*p* < 0.001 vs. EAM.

Cardiac apoptosis is a common mechanism of cardiomyocyte damage in severe human myocarditis of various etiologies and histopathologic presentations [38] as well as in EAM [39]. On the other hand, PKC has been shown to exert both inhibitory and stimulatory influences on apoptosis [40]. Therefore, to further investigate the molecular mechanism of the protective role of Ro-32-0432 on the heart of the EAM rats, two major pathways for apoptosis were explored, including the death receptor-induced extrinsic pathway and the mitochondria-mediated apoptotic intrinsic pathway [41]. At week 3 of EAM, the mRNA level of anti-apoptotic member (Bcl-2) was down-regulated from the control group of 1.13 ± 0.19 to 0.13 ± 0.04 for the EAM group, while rats treated with Ro-32-0432 showed lesser reduction (0.33 ± 0.06) (Figure 6A). In contrast, the mRNA level of pro-apoptotic member (Bax) was up-regulated from the control of 1.08 ± 0.21 to 2.84 ± 0.39 for the EAM, and slightly down-regulated to 2.19 ± 0.16 by Ro-32-0432 treatment (Figure 6B). Thus, the ratio of Bax/Bcl-2 was reversed by Ro-32-0432 (Figure 6C), implying an anti-apoptotic role of Ro-32-0432. Furthermore, the relative protein expression level of cleaved caspase-3, which is the activated form of caspase-3 and acts as a lethal protease at the most distal stage of the apoptosis pathway [42], was significantly increased from 0.16 ± 0.01 in control group to 0.60 ± 0.09 in the EAM group (Figure 6D and 6E), while the protein level of full length caspase-3 was not significantly changed (Figure 6D). Consistent with its effect on Bax/Bcl-2, Ro-32-0432 decreased the relative protein level of cleaved caspase-3 to 0.22 ± 0.03 (Figure 6D and 6E). On the other hand, EAM-induced a significant increase in relative mRNA level of the death receptor Fas; but application of Ro-32-0432 had no significant effect on the Fas mRNA level (Figure 6F). These results suggest that PKC inhibitor suppresses EAM-induced apoptosis of myocardial cells through the intrinsic apoptotic pathway.

### PKC inhibitor suppresses EAM induced cardiac fibrosis

**Figure 7:**
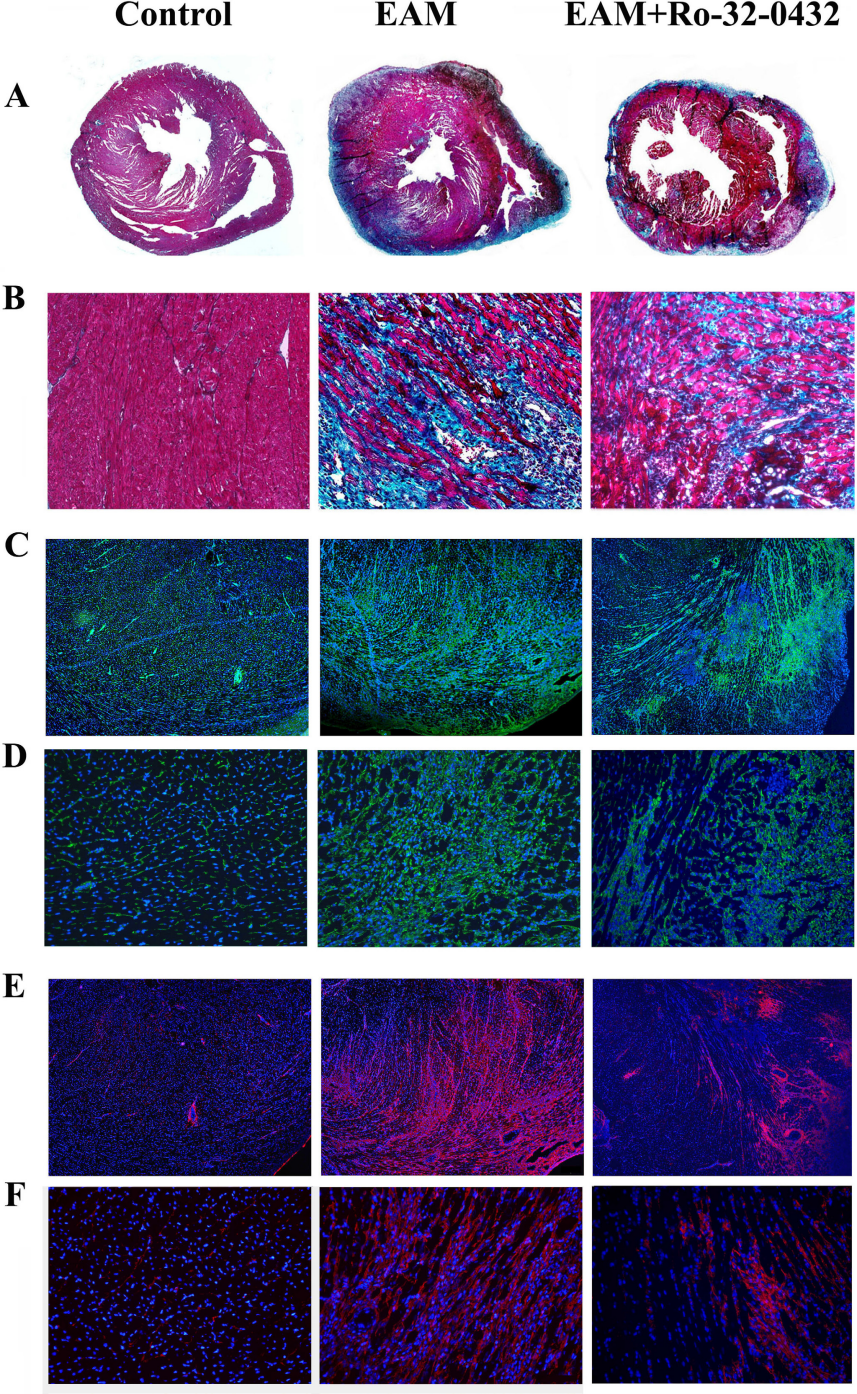
Ro-32-0432 reduces cardiac fibrosis in EAM rats (**A** and **B**) Representative masson trichrome-stained sections showing cardiac fibrosis (blue). (**C**–**F**) Immunofluorescence staining of vimentin (C and D) and collagen type I (E and F) for control, EAM and Ro-32-0432 treated EAM groups. A, C and E: magnification 4x; B, D and F: magnification 20x. green: vimentin; red: collagen type I; blue: DAPI stained nuclei.

It has been shown that PKC-α is involved in cardiac fibrosis [43] and its inhibition could reduce ventricular fibrosis [44]. Therefore, we investigated the effect of Ro-32-0432 on EAM-induced cardiac fibrosis. Histological study revealed that cardiac fibrosis was highly increased in the EAM rat and reduced by Ro-32-0432 treatment (Figure 7A and 7B). Furthermore, immunofluorescence staining showed that vimentin (Figure 7C and 7D) and collagen type I expression (Figure 7E and 7F) were upregulated in the heart of EAM rat, indicating an increased fibroblast proliferation. Ro-32-0432 treatment inhibited both vimentin (Figure 7C and 7D) and collagen type I expression in the cardiac tissue of EAM rat (Figure 7E and 7F). In contrast, no significant fibrosis area, and much less vimentin and collagen type I expression were found in normal rats. These results suggest that PKC inhibitor suppresses EAM induced cardiac fibrosis.

### PKC inhibitor partially suppresses EAM-induced cardiac inflammation

**Figure 8:**
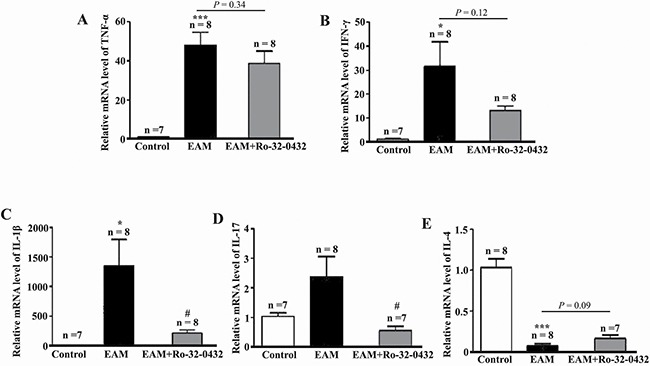
Ro-32-0432 suppresses key inflammatory cytokines expression in the heart of EAM rats (**A**–**E**) Relative mRNA expression levels of TNF-α (A), IFN-γ (B), IL-1β (C), IL-17 (D) and IL-4 (E) in the heart of control, EAM and Ro-32-0432 treated EAM groups. *p < 0.05 vs. control; ***p < 0.001 vs. control; ^#^*p <* 0.05 vs. EAM.

EAM is a T cell-mediated inflammatory disorder of cardiac muscle [45]. To determine whether Ro-32-0432 affects EAM-induced cardiac inflammation, we assessed the impact of Ro-32-0432 treatment on the mRNA expression of Th1, Th2 and Th17 associated cytokines in the heart of EAM rats. Comparing to the control group, Th1 cytokines TNF-α (Figure 8A), IFN-γ (Figure 8B) and Th17 cytokine IL-17 (Figure 8D) as well as Th17 differentiation regulator cytokine IL-1β (Figure 8C) were significantly increased in EAM rats. Meanwhile, the level of Th2 cytokine IL-4 was significantly decreased (Figure 8E). Intriguingly, Ro-32-0432 treatment only significantly reduced EAM-induced expression of IL-17 (Figure 8D) and IL-1β (Figure 8C) without significant effect on the expression of IL-4, IFN-γ and TNF-α. These results indicate that Ro-32-0432 could partially down-regulate proinflammatory cytokines IL-1β and IL-17 in EAM.

## DISCUSSION

One potential issue associated with the data presented here is the specificity of Ro-32-0432. Ro-32-0432 is a systemically administrable highly selective inhibitor of PKC with much higher selectivity for PKC compared with other protein kinases, such as PKA, myosin light chain kinase, casein kinase II and tyrosine-specific protein kinases [46]. In addition, amongst the PKC isoforms, Ro-32-0432 displays higher selectivity for Ca^2+^-dependent PKC isoforms such as PKC-α, compared with novel Ca^2+^-independent isoenzymes such as PKCε [46, 47]. Therefore, as a pan-PKC inhibitor, we could not completely exclude the possibility that Ro-32-0432 took the action by inhibiting other PKC isoforms. However, the following pieces of evidence suggest that Ro-32-0432 may take the action by mainly inhibiting PKC-α in EAM rats. First, PKC-α is the dominant PKC isozyme expressed in the heart [21, 48, 49]. Second, even though many other PKC isoforms, such as PKC-β, PKC-δ and PKC-ε are expressed in the heart, in our model of EAM, their mRNA expression levels were not significantly increased. Third, EAM not only induced increase in the mRNA level and protein level of PKC-α, but also induced upregulation of PKC-α activity. Finally, Ro-32-0432 has been determined to be more potent for inhibiting PKC-α than for other PKC isoforms [21].

Apoptosis has been shown to play key roles in the pathogenesis in a variety of cardiovascular diseases [50, 51]. Since cardiac myocytes are terminally differentiated and not replaced after they are lost, loss of cardiac myocytes via apoptosis has important pathophysiological consequences, contributing to the loss and functional abnormalities of the myocardium and to the continuous decline of ventricular function in heart failure. Therefore, prevention of cardiomyocyte loss in cardiovascular diseases is critical to prevent development of heart failure. In the heart, the death receptor-induced extrinsic pathway and the mitochondria-mediated apoptotic intrinsic pathway are the two major pathways for apoptosis of myocardial cells [41]. In our study, we show that EAM not only induced increase in Bax/Bcl-2 and protein expression level of cleaved caspase-3, but also induced up-regulation of the death receptors Fas mRNA level, suggesting that both intrinsic and extrinsic apoptotic pathways are involved in the pathogenesis of myocarditis. However, Ro-32-0432 only affected the intrinsic apoptotic pathway, but had no effect on the extrinsic apoptotic pathway. This result implies that combination of inhibitors for both PKC and the death receptor signaling might be more effective to alleviate EAM.

During the initiation of EAM, naïve CD4^+^ T-helper (Th) cells differentiate into IFNγ-producing Th1, IL-4-producing Th2 and IL-17-producing Th17 cell subsets [52, 53]. It has been demonstrated that the Th1/Th2 imbalance plays crucial roles in the induction and progression of EAM [54, 55]. In agreement with this notion, our results showed that EAM induced increase in IFN-γ but reduction of IL-4. However, Ro-32-0432 had no significant effect on the expression of Th1 cytokines IFN-γ, TNF-α and Th2 cytokine IL-4. On the other hand, Th17 cells have been proposed to participate in the pathogenesis of myocarditis [56, 57]. Our study showed that Ro-32-0432 reduced the expression of Th17 cytokine IL-17 and Th17 differentiation regulator cytokine IL-1β in EAM rat, suggesting that Ro-32-0432 may suppress myocardial inflammatory response by inhibiting Th17 differentiation.

It has been indicated that multiple PKC isozymes are expressed in all cell types throughout the body and have crucial roles in normal physiology. As a result, inhibition of PKC may result in undesired side effects [24]. However, recent clinical trials suggest that systemic delivery of inhibitors and activators of PKC isozymes is well tolerated [58]. Furthermore, PKC-α knockout mice were born at the predicted mendelian frequencies with normal blood pressure and heart rate, and were overtly normal for up to 16 months [10], suggesting that even lack of PKC-α is well tolerated. In consistence with this inference, both systemic safety assessments and ocular safety assessments indicated that ruboxistaurin, an inhibitor of both PKC-β and PKC-α, is well tolerated in patients with diabetes [59, 60]. Besides, it has been suggested that targeting enzymes that are in the middle of the signaling cascade, such as PKC isozymes, is more suitable for drug development, because early events in signal transduction pathways often include side branches, amplification steps, and feedback loops [61]. In this report, we show that PKC inhibitor reduces the EAM induced expression of failure biomarkers, suppresses myocarditis induced apoptosis of myocardial cells and some proinflammatory cytokines levels associated with myocarditis. Even though inhibition of PKC has been well studied in various cardiovascular diseases, the effect of PKC inhibitor on myocarditis has not been studied yet. Therefore, considering that myocarditis is still one of the most challenging diseases to treat in cardiology, it is worth to explore the possibility of using PKC inhibitor to treat myocarditis.

## MATERIALS AND METHODS

### Induction of EAM model

Male Lewis rats (180–200g), aged 6–8 weeks, purchased from Beijing Vital River Laboratory Animal Technology (Beijing, China) were maintained in Xiamen University animal experiment center. Cardiac myosin was prepared from the ventricular muscle of porcine hearts as previously described [62]. To produce EAM, each rat was immunized on day 0 with 0.2 mL emulsion containing 1 mg cardiac myosin with an equal volume of complete Freud's adjuvant supplemented with mycobacterium tuberculosis H37RA at a concentration of 10 mg/mL by a single subcutaneous injection in both footpads [62]. The rats in the control group were only immunized with complete Freund's adjuvant. All animal care and experiments were performed in accordance with procedures approved by the Animal Care and Use Committee of Xiamen University. The relative mRNA and protein levels were measured at week 3 of EAM.

### *In vivo* administration of Ro-32-0432 to EAM rats

Ro-32-0432 was dissolved in DMSO at a concentration of 3 mg/ml. It was intraperitoneally injected every two days at a dosage of 1 mg/kg to the rats from day 14 to day 18 of EAM.

### Histopathological analyses

All rats were sacrificed at week 3. The heart weight (HW) and body weight (BW) were measured to calculate HW/BW. Ten percent formalin-fixed and paraffin-embedded cardiac ventricles were cut into 5-μm-thick transverse sections for hematoxylin-eosin (HE) staining to evaluate the histopathology of the heart [62]. Macroscopic scores were classified into five grades [63]: 0, no inflammation; 1, presence of a small discolored focus; 2, presence of multiple small discolored foci; 3, diffuse discolored areas not exceeding a total of one third of the cardiac surface; and 4, diffuse discolored areas totaling more than one third of the cardiac surface. In order to calculate histoscore, the severity of myocarditis induced inflammation in the cardiac cross sections was graded as described previously [63]: 0, no inflammation; 1, presence of a few small lesions, not exceeding 0.25 mm^2^ in size; 2, presence of multiple small lesions or a few moderately sized lesions, not exceeding 6.25 mm^2^; and 3, presence of multiple moderately sized lesions or more, larger lesions. Cardiac fibrosis was detected with Masson Trichrome staining kit (KGMST-8003; keygen, China). All histopathological analyses were performed at week 3 of EAM by an individual blinded to treatment groups.

### Enzyme-linked immunosorbent assay (ELISA)

Blood samples were collected from inferior vena cava. Rat serum was separated after centrifugation of blood samples at 3,000 rpm for 20 min and the serum cTNT and BNP level was measured by ELISA kit according to the manufacturer's instructions (Aviva systems biology, USA). Briefly, standards, serum samples or blank were added to each well and incubated at 37°C for 1 hour. After aspirated and washed. A biotinylated detector antibody was added to each well and incubated for 30 minutes, followed by washes. Then, avidin-HRP conjugate was added to each well and incubated for 30 minutes. After aspirated and washed, TMB substrate was added to each well and incubated in the dark for 10 minutes. Then, stop solution was added to each well, followed by detecting the O.D. absorbance at 450nm with a microplate reader within 5 minutes.

### RNA isolation and real-time PCR

Total RNA of cardiac ventricles was extracted using Trizol reagent (Life technology, USA) following manufacturer's instructions. Synthesis of cDNA was performed using a RevertAid First Strand cDNA Synthesis Kit (Thermo, Lithuanie). The primers (Table 1) were synthesized by Takara Bio Inc (Dalian, China) according to cDNA sequences. Real-time PCR was carried out in the CFX96 Real-Time System using SYBR Green detection reagent (TOYOBO, Japan). The relative mRNA levels were calculated by using 2^–ΔΔCt^ method [64] and normalized against glyceraldehyde-3-phosphate dehydrogenase (GAPDH).

**Table 1:** Primer sequences of qPCR

Gene	sense primer	Antisense primer
ANP	5′-atggatttcaagaacctgctaga-3′	5′-gctccaatcctgtcaatcctac-3′
BNP	5′-gatgattctgctcctgcttttc-3′	5′-gccatttcctctgacttttctc-3′
IL-1β	5′-gctagtgtgtgatgttcccattag-3′	5′-cttttccatcttcttctttgggta-3′
TNF-α	5′-atgggctccctctcatcagt-3′	5′-actccagctgctcctctgct-3′
IFN-γ	5′-aggccatcagcaacaacataagtg-3′	5′-gacagclttgtgctggatclgtg-3′
IL-4	5′-accttgctgtcaccctgttc-3′	5′-ttgtgagcgtggactcattc-3′
IL-17	5′-tactcatccctcaaagttcagtgt-3′	5′-ctcttgctggatgagaacagaat-3′
Bcl-2	5′-aggattgtggccttctttgagtt-3′	5′-gccggttcaggtactcagtcat-3′
Bax	5′-ttgctgatggcaacttcaactg-3′	5′-ctttagtgcacagggccttgag-3′
Fas	5′-ctgcagatatgctgtggatca-3′	5′-tttggtgttgctggttggt-3′
PKC-α	5′-ttcccaatcatcatagcaca-3′	5′-gagatagttatcaaccgagcag-3′
PKC-β2	5′-gaactgactcccactgaca-3′	5′-caccatgaatcctgga-3′
PKC-δ	5′-gttcatcgccaccttctttg-3′	5′-atttcttatggatggcagcg-3′
PKC-ε	5′-gcgaagcccctaagacaat-3′	5′-caccccagatgaaatccctac-3′

### Immunofluorescence analysis

5-μm cryostat sections of hearts were fixed in 4% paraformaldehyde for 10 min, and processed for immunofluorescence staining as previously described [65]. The sections were blocked by 10% bovine serum albumin for 30 min and stained with vimentin (1:50, abcam) or collagen type I (1:50, boster) for 1 hour at 37°C. Afterwards, the sections were washed 3 times in PBS and incubated with Alexa 488 or 549-conjugated goat anti-rabbit secondary antibodies (Rockland) for 1 h at room temperature. Nuclei were stained with DAPI. Slices were mounted with antifade mounting medium (Applygen, China) and analyzed using Olympus microscope (IX51).

### Extraction of membrane and cytosolic proteins

Ventricular tissues were lysed in cold extraction buffer (0.32 M sucrose, 5 mM Tris-HCL, 120 mM KCL, 1 mM EGTA, 1 mM EDTA, 1 mM PMSF, 1 mM leupeptin, pH 7.5) for 30 min on ice. Isolation of membrane and cytosolic fraction of proteins was carried out as previously described [66]. The homogenates were centrifuged at 100,000 g for 1 h at 4°C. The supernatants were used for western blot analysis of the cytoplasmic protein. The pellets were dissolved in 2% TritonX-100 buffer (20 mM HEPES, 10% glycerol, 1 mM EDTA, 1 mM EGTA, 1 mM PMSF, 1 mM leupeptin, pH 7.5) for 1 hour on ice. Then, it was centrifuged at 12,000 g for 1 h at 4°C. The supernatants were used for western blot analysis of the membrane protein.

### Western blot analysis

The cardiac ventricles were lysed in RIPA buffer (Product Code: P0013B, Beyotime, China) in the presence of 1 mM phenylmethanesulfonyl fluoride (Solarbio, China) and protein phosphatase inhibitor complex I (Aidlab, China), which contains sodium orthovanadate, sodium fluoride, sodium molybdate, sodium tartrate dihydrate and imidazole. Protein samples were separated by 10% SDS-PAGE gels and transferred to PVDF membranes (Millipore, USA). The membranes were blocked by 5% non-fat milk for 1 h and incubated with anti-caspase 3 antibody (Cell Signaling; 1:1000), anti-β-actin antibody (Immunoway; 1:2000), anti-PKC-α antibody (abcam; 1:4000), anti-GAPDH antibody (Epitomics; 1:5000). After washing with PBST for 5 times, the membranes were incubated with goat anti-rabbit antibody (Sigma; 1:10000) for 1 h at room temperature. Densitometry of bands was analyzed using Image J software (National Institutes of Health, USA).

### Statistical analysis

All data were shown as mean ± SEM. The comparison of histopathological scores was evaluated using Mann-Whitney *U*-test (SPSS 13.0 Software). Comparisons of other data were carried out with one-way analysis of variance (ANOVA) using Prism 5.0 (GraphPad Software, Inc) software. Values of *p* < 0.05 were considered to be statistically significant.

## Abbreviations

EAM: experimental autoimmune myocarditis
ANP: atrial natriuretic peptide
BNP: brain natriuretic peptide
